# A Phase 3 Safety and Efficacy Study of Baloxavir Marboxil in Children Less Than 1 Year Old With Suspected or Confirmed Influenza

**DOI:** 10.1097/INF.0000000000004826

**Published:** 2025-04-28

**Authors:** Sauli Palmu, Larisha Pillay-Ramaya, Jeffrey Baker, Katherine Kocsis, Manisha Kanwar, Eriola Berisha, Steffen Wildum, Laura Burleigh Macutkiewicz, Mercedes Macías Parra

**Affiliations:** From the * Tampere University Hospital, Department of Pediatrics, and Tampere University, Faculty of Medicine and Health Technology, Center for Child, Adolescent and Maternal Health Research, Tampere, Finland; † Global Clinical Trials, Pretoria, South Africa; ‡ Clinical Research Prime, Idaho Falls, Idaho; § Roche Products Ltd, Welwyn Garden City, United Kingdom; ¶ F. Hoffmann-La Roche Ltd, Mississauga, Canada; ‖ F. Hoffmann-La Roche Ltd, Basel, Switzerland; ** Instituto Nacional de Pediatría, Mexico City, Mexico.

**Keywords:** baloxavir marboxil, influenza, pediatrics, clinical trial, miniSTONE-1

## Abstract

**Background:**

Baloxavir marboxil (baloxavir) inhibits influenza virus cap-dependent endonuclease and has demonstrated safety and efficacy in children 1–<12 years of age. This study assessed the safety and efficacy of baloxavir in children <1 year old.

**Methods:**

miniSTONE-1 (NCT03653364) was a Phase III, global, multicenter, single-arm study to evaluate patients <1 year of age who received a single dose of baloxavir (age ≥3 months: 2 mg/kg; <3 months 1 mg/kg). The primary endpoint was safety; secondary endpoints included pharmacokinetics and efficacy (time to alleviation of signs and symptoms, duration of fever and symptoms, antibiotic use and cessation of viral shedding).

**Results:**

Overall, 48/49 enrolled patients received baloxavir, of whom 15 had positive centralized influenza reverse transcription polymerase chain reaction tests and comprised the intent-to-treat influenza-infected population. The median age was 6 months and 79.2% of patients were not influenza-vaccinated. Overall, 51 adverse events (AEs) were reported in 23 patients; most were grade 1–2. The most common AEs were diarrhea (16.7%) and vomiting (12.5%). Two patients experienced serious AEs unrelated to treatment. In the intent-to-treat influenza-infected population, median time to alleviation of signs and symptoms was 163.7 hours [95% confidence interval (CI): 122.5–not estimable], median duration of fever was 23.1 hours (95% CI: 22.3–44.6) and median time to cessation of viral shedding was 24.5 hours (95% CI: 24.2–68.6).

**Conclusions:**

Baloxavir was well tolerated in children <1 year of age, with no new safety signals identified. Clinical, virological and safety outcomes were consistent with established profiles in adults, adolescents and children 1–<12 years old.

Influenza is a contagious respiratory illness that infects as many as 1 billion people each year.^1^ Children have an increased risk of hospitalization as a result of influenza infection compared with other age groups,^2^ and children <1 year old face a particularly high risk, owing to their lack of acquired protective antibodies.^3,4^ Furthermore, influenza vaccination is not recommended for children <6 months of age.^5^ Historically, each winter season, up to 10% of children <1 year of age in the United States (USA) require medical attention for influenza-related illnesses^4^ and, worldwide, approximately 374,000 hospitalizations are attributed to influenza infections in this age group.^4,6^

Antiviral treatment options for influenza in children <1 year old are limited. In the USA, the neuraminidase inhibitor oseltamivir is approved for use in children ≥2 weeks of age; for treatment of influenza, the dosing regimen is twice-daily oral administration for 5 days.^7^ Therefore, there is a degree of treatment burden associated with oseltamivir administration. In a Phase I/II clinical study of oseltamivir in patients <1 year old, the most common adverse events (AEs) were vomiting and diarrhea, and the majority of gastrointestinal AEs were unrelated to treatment.^8^ Overall, gastrointestinal disorders are the most common class of adverse reactions reported for oseltamivir use across age groups, and vomiting is the most commonly reported in children.^8,9^ Intravenous zanamivir and peramivir are additional treatment options for influenza in children ≥6 months of age in the EU and USA, respectively.^10–12^ However, the intravenous route of administration can be challenging for many patients, particularly those with poor venous access, such as infants.^13,14^ Intravenous peramivir is not commonly used in the outpatient setting,^15^ and intravenous zanamivir is only approved for use in patients with complicated influenza who require hospitalization.^10^ In addition, these treatment options are less widely available compared with generic oseltamivir products.^16^ Consequently, there is an unmet need for an antiviral treatment option that is effective and well tolerated in children <1 year of age, with a practical route of administration and a simple dosing regimen.

Baloxavir marboxil (baloxavir) is an inhibitor of the influenza virus cap-dependent endonuclease, with enhanced ease of administration due to its oral formulation and single-dose regimen, which is particularly beneficial to infants. Baloxavir has demonstrated efficacy for the treatment and postexposure prophylaxis of uncomplicated influenza in adults and children ≥1 year old.^17–20^ In a double-blind, randomized, active-controlled study (miniSTONE-2), baloxavir was found to be both well-tolerated and effective at alleviating symptoms of acute influenza in children 1–<12 years of age.^20^ miniSTONE-1 (NCT03653364), a single-arm, open-label study, was designed to assess the safety, pharmacokinetics (PK) and efficacy of single-dose baloxavir in patients < 1 year of age. Here, we report the safety and efficacy results from miniSTONE-1; PK data will be reported separately.

## METHODS

### Study Design and Participants

miniSTONE-1 was a global, multicenter, single-arm study evaluating the safety, PK and efficacy of single-dose oral baloxavir in patients <1 year old. The study was conducted at 15 centers across 7 countries (Costa Rica, Finland, Mexico, Poland, South Africa, Spain and the USA) between January 2019 and June 2023. Patients were initially eligible to enroll in the study based on the presence of symptomatic evidence of influenza (respiratory symptoms and temperature). A global protocol amendment was made on June 1, 2020, because of the coronavirus disease 2019 (COVID-19) pandemic, in which patients with clinically suspected influenza and at least one respiratory symptom (either cough or coryza) were also required to have a positive influenza test [rapid influenza diagnostic test or reverse transcription polymerase chain reaction (RT-PCR)] and negative COVID-19 test within 48 hours of screening. Patients were excluded if they were hospitalized for complications of influenza, or if they had significant co-morbidities (concurrent infections that required systemic antiviral therapy; known HIV infection or an immunosuppressive disorder; uncontrolled renal, vascular, neurologic or metabolic disease; hepatitis; cirrhosis; pulmonary disease; chronic renal failure; active cancer at any site; or a history of organ transplantation). Preterm neonates (born at <37 weeks gestation), patients who weighed <2.5 kg and patients who received immunization with a live/attenuated influenza vaccine within 2 weeks before screening were also excluded.

### Treatment

All patients received a single dose of baloxavir as granules for oral suspension (reconstituted with drinking water) on Day 1 only. Dose was based on age (Cohort I: ≥3 months–12 months of age, 2 mg/kg; Cohort II: ≥4 weeks–<3 months, 1 mg/kg; Cohort III: birth–<4 weeks, 1 mg/kg); doses were chosen based on population PK analyses and aimed to match adult PK exposures. Patients were followed up to Day 29, with mandatory clinic visits on Days 2, 4, 6, 10 and 29.

### Outcomes Measured

The primary endpoint was safety, defined as the percentage of patients with AEs and serious AEs (SAEs) up to Day 29. Secondary endpoints comprised PK and clinical and virological efficacy. Clinical efficacy endpoints included time to alleviation of signs and symptoms (TTAS) of influenza, defined as time taken from the start of treatment to the point at which all of the following criteria were met and sustained for at least 21.5 hours: a score of 0 (no problem) or 1 (minor problem) was reported for cough and nasal symptoms on the Canadian Acute Respiratory Illness and Flu Scale questionnaire; a return to normal daily activity; and a return to an afebrile state (tympanic temperature ≤37.2 degrees). A sensitivity analysis for TTAS was performed, in which the return to normal daily activity criterion was removed. Duration of fever and duration of symptoms up to Day 15, and frequency of influenza-related complications (death, hospitalization, radiologically confirmed pneumonia, bronchitis, sinusitis, otitis media, encephalitis/encephalopathy, febrile seizures and myositis) and proportion of patients requiring antibiotics for influenza-related complications up to Day 29, were assessed. PK parameters including plasma concentrations, half-life, maximum plasma concentration and time to maximum plasma concentration were also assessed, and results will be reported in a separate publication. Virological efficacy endpoints included time to cessation of viral shedding by viral titer and change from baseline in influenza viral titer at Days 2, 4, 6, 10 and 29, as assessed by tissue culture infectious dose (TCID_50_).

Exploratory virological endpoints included the frequency and genotypes (assessed by Sanger sequencing with a lower limit of detection of 4.5 log10 virus particles per mL) of viruses with polymerase acidic (PA) gene mutations potentially conferring resistance to baloxavir. Phenotypic analysis to assess baloxavir susceptibility of a novel PA/T20K variant was performed in vitro using a recombinant influenza B virus and the ViraDot assay, as previously described.^21^ Susceptibility to baloxavir was determined by measuring the half-maximal effective concentration (EC_50_); reduced susceptibility was defined as a fold-change in EC_50_ of >10 for influenza A and >5 for influenza B.

### Statistical Analysis

The primary endpoint for this study was evaluated in the safety population, which comprised patients who received 1 dose of baloxavir. The intent-to-treat influenza-infected (ITTi) population was used for efficacy analysis, defined as patients with confirmed influenza infection by centralized RT-PCR testing.

As this was a single-arm study, no formal statistical hypothesis testing or sample size calculations were performed. All outcomes were summarized descriptively.

### Ethics and Consent

This study was conducted in accordance with the Declaration of Helsinki and the International Council for Harmonization Good Clinical Practice (ICH E6) guidelines^22,23^ and was approved by all relevant institutional review boards and/or ethics committees at each center. All parents/caregivers of participants gave written informed consent to participate in the study.

## RESULTS

### Patient Population

**FIGURE 1.**
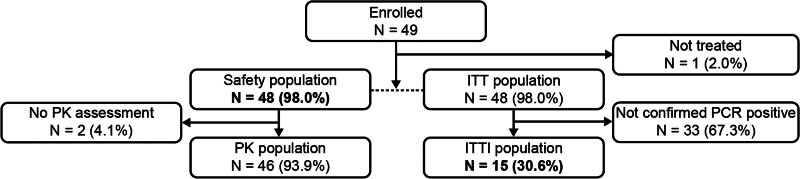
Patient disposition. ITT indicates intent-to-treat; ITTi, intent-to-treat influenza-infected; PCR, polymerase chain reaction; PK, pharmacokinetics.

A total of 49 patients were enrolled between January 2019 and July 2023, of whom 48 received baloxavir and 46 completed the study (Fig. 1). Most patients were recruited in South Africa (n = 25) and the USA (n = 13). Forty-three patients were recruited based on symptomatic evidence of influenza, without the requirement for a positive influenza test or a negative COVID-19 test; 6 patients were recruited after this requirement was introduced by a protocol amendment. Of the patients who received treatment (n = 48), the median age at baseline was 6 months (26.3 weeks). Most patients (39/48 [81.3%]) were 3 to <12 months of age (Cohort I); 8 patients (16.7%) were 4 weeks to <3 months (Cohort II) and 1 patient (2.1%) was <4 weeks old (Cohort III). Approximately half of patients (26/48 [54.2%]) were Black or African American and most patients (38/48 [79.2%]) were not vaccinated against influenza. No patients were enrolled into this study with disorders that may otherwise have been considered high risk for complications of influenza. Of the 15 patients who had an influenza infection confirmed by RT-PCR, influenza A/H3 was the most common subtype (7/15, 46.7%). Demographics and baseline characteristics are detailed in Table 1.

**TABLE 1.** Demographics and Baseline Characteristics in the Safety Population

	Baloxavir (N = 48)
Age (weeks)	
n	48
Mean (SD)	29.5 (15.2)
Median (Min–Max)	26.3 (3.3–52.1)
Age group, n (%)	
n	48
Cohort I: ≥3–12 months	39 (81.3)
Cohort II: ≥4 weeks–<3 months	8 (16.7)
Cohort III: Birth–<4 weeks	1 (2.1)
Ethnicity, n (%)	
n	48
Hispanic or Latino	15 (31.3)
Not Hispanic or Latino	22 (45.8)
Unknown	9 (18.8)
Not reported	2 (4.2)
Race, n (%)	
n	48
American Indian or Alaska Native	1 (2.1)
Black or African American	26 (54.2)
White	21 (43.8)
Vaccination status, n (%)	
n	48
Yes	10 (20.8)
No	38 (79.2)
Influenza type/subtype by RT-PCR, n (%)	
n	15
B	2 (13.3)
A/H1N1pdm09	5 (33.3)
A/H3	7 (46.7)
A/Unknown	1 (6.7)
Influenza season, n (%)*	
n	48
2018/2019 Northern Hemisphere	3 (6.3)
2019 Southern Hemisphere	30 (62.5)
2019/2020 Northern Hemisphere	9 (18.8)
2020 Southern Hemisphere	0
2020/2021 Northern Hemisphere	0
2021 Southern Hemisphere	1 (2.1)
2021/2022 Northern Hemisphere	1 (2.1)
2022 Southern Hemisphere	1 (2.1)
2022/2023 Northern Hemisphere	3 (6.3)
2023 Southern Hemisphere	0

* Influenza season for the Northern Hemisphere was October 4 to April 3; influenza season for the Southern Hemisphere was April 4 to October 3.

RT-PCR indicates reverse transcription polymerase chain reaction; SD, standard deviation.

### Safety

Within the safety population (n = 48), a total of 51 AEs were reported in 23 (47.9%) patients. The most common AEs were diarrhea (8/48 [16.7%]) and vomiting (6/48 [12.5%]; Table 2), which was also observed in the ITTi population, with diarrhea and vomiting reported in 5/15 (33.3%) and 4/15 (26.7%) patients, respectively. The majority of AEs were grade 1 or 2 in severity; 2 patients (4.2%) experienced grade 3 events (lower respiratory tract infection and decreased lymphocyte concentration) and 1 patient (2.1%) experienced a grade 5 event (choking). Two patients (4.2%) experienced a nonserious AE thought to be related to baloxavir (both grade 1 diarrhea). SAEs occurred in 2 patients (4.2%), both considered unrelated to treatment (lower respiratory tract infection (grade 3) and choking [grade 5]). There were no clinically meaningful observed changes in vital signs or Hy’s law events.

**TABLE 2.** Adverse Events Reported According to System Organ Class in the Safety Population

	Baloxavir (N = 48)
Patients with ≥1 AE, n (%)	23 (47.9)
Total number of AEs	51
SAEs	2 (4.2)*
Death	1 (2.1)
AEs by SOC (most commonly reported in patients) and preferred term, n (%)	
Gastrointestinal disorders	
Total number of patients	11 (22.9)
Total number of events	22
Diarrhea	8 (16.7)
Vomiting	6 (12.5)
Infections and infestations	
Total number of patients	9 (18.8)
Total number of events	10
Genital candidiasis	2 (4.2)
Lower respiratory tract infection	2 (4.2)
Otitis media	2 (4.2)
Respiratory, thoracic and mediastinal disorders	
Total number of patients	7 (14.6)
Total number of events	10
Cough	2 (4.2)
Nasal congestion	2 (4.2)
Productive cough	2 (4.2)

* SAEs included: grade 3 lower respiratory tract infection and grade 5 choking. Both events were considered unrelated to the study treatment by the investigator.

AE indicates adverse event; SAE, serious adverse event; SOC, system organ class.

### Clinical Outcomes

In the ITTi population (n = 15), the median TTAS based on the Canadian Acute Respiratory Illness and Flu Scale questionnaire was approximately 7 days (163.7 hours [95.0% confidence interval (CI): 122.5–not estimable]; Table 3). In a sensitivity analysis, which removed the criterion for return to normal daily activity, TTAS was approximately 4 days (98.2 hours [95.0% CI: 49.7–161.8]; n = 15), which was numerically lower than in the main analysis (Table 3).

**TABLE 3.** Overview of Clinical Efficacy Endpoints in the Intent-to-treat Influenza-infected Population

	Baloxavir (N = 15)
Time to alleviation of influenza signs and symptoms	
Patients included in this analysis	15
Patients with event, n (%)	9 (60.0)
Median time to event, hours (95% CI)	163.7 (122.5–NE)
Sensitivity analysis for time to alleviation of influenza signs and symptoms*	
Patients included in this analysis	15
Patients with event, n (%)	13 (86.7)
Median time to event, hours (95% CI)	98.2 (49.7–161.8)
Duration of fever (return to afebrile state)	
Patients included in this analysis	13†
Patients with event, n (%)	13 (100.0)
Median duration, hours (95% CI)	23.1 (22.3–44.6)
Duration of symptoms	
Patients included in this analysis	15
Patients with event, n (%)	8 (53.3)
Median duration, hours (95% CI)	163.7 (71.0–NE)
Time to return to normal health and activity	
Patients included in this analysis	14
Patients with event, n (%)	9 (64.3)
Median time to event, hours (95% CI)	140.7 (72.2–NE)
Frequency of influenza-related complications	
Patients included in this analysis	15
Patients with event, n (%)	0 (0.0)
Proportion requiring antibiotics‡	
Patients included in this analysis	15
Patients, n (%)	0 (0.0)

* The sensitivity analysis removed the criterion for return to normal activity.

† Patients who did not have fever at baseline or whose body temperature was not collected were excluded from this analysis.

‡ Proportion of patients who required antibiotics only included patients who required antibiotics for influenza-related complications.

CI indicates confidence interval; NE, not estimable.

The median duration of fever was approximately 1 day (23.1 hours [95.0% CI: 22.3–44.6], n = 13) and the median duration of symptoms was approximately 7 days (163.7 hours [95.0% CI: 71.0–not estimable]; n = 15). There were no influenza-related complications and no patients requiring antibiotics for influenza-related complications in the ITTi population (Table 3).

### Virologic Outcomes

Of the patients with postbaseline viral titer assessment (n = 10), the median time to cessation of viral shedding by viral titer was approximately 1 day (24.5 hours [95.0% CI: 24.2–68.6]). The mean change in influenza viral titer from baseline (mean [standard deviation], log_10_ TCID_50_/mL) at Days 2, 4, 6, 10 and 29 was −2.50 (1.70), −2.60 (1.95), −2.43 (1.40), −2.83 (1.81) and −1.69 (1.71), respectively. Of the 13 patients with paired samples for sequencing analysis (pre- and post-dosing with baloxavir) or at least 1 postdose sample, the observed frequency of treatment-emergent resistance was 15.4% (2/13; 1 PA/I38T/I mutation [influenza A/H1N1pdm09] and 1 PA/T20K/T mutation [influenza B]). The patient with a PA/I38T/I mutation achieved first cessation of viral shedding after 3.1 days (75.3 hours), but exhibited viral titer rebound on Day 6; the TTAS for this patient was 3.0 days (72.2 hours). The patient with a PA/T20K/T substitution achieved the first cessation of viral shedding after 2.6 days (63.1 hours), without viral titer rebound; the TTAS for this patient was 3.7 days (89.0 hours). PA/T20K is a novel amino acid substitution, which was confirmed in vitro to reduce baloxavir susceptibility of influenza B virus by approximately 7-fold (EC_50_ 23.92 ± 2.94 nM [SD]).

## DISCUSSION

Baloxavir is a cap-dependent endonuclease inhibitor that inhibits viral replication at an early stage.^13^ The safety and efficacy of single-dose baloxavir for treatment of influenza has been demonstrated in adults, adolescents and children (1–<12 years of age).^17,18,20^

This report describes the safety and efficacy results of a Phase III, single-arm, open-label study of baloxavir in patients <1 year old, a patient population with limited treatment options. These results expand on existing data from 2 Japanese open-label studies, which included a small number of patients <1 year old and demonstrated baloxavir to be well tolerated with a rapid reduction in viral load associated with symptom alleviation.^24,25^

The primary endpoint of this study was safety. The most common AEs were diarrhea (16.7%) and vomiting (12.5%). The safety profile observed for patients <1 year of age in this study was similar to that previously reported in older children and adults.^17,18,20^ A single dose of baloxavir was well tolerated in this patient population; no new safety signals were identified and most AEs were nonserious, low grade and considered unrelated to the study treatment. Clinical and virological endpoints were generally consistent with established profiles in patients 1–<12 years old, with a short median duration of fever and rapid cessation of viral shedding.^20^

All influenza antivirals exert selective pressure on viruses, which can lead to the emergence of variants with reduced susceptibility.^26^ In this study, the resistance rate in patients <1 year of age (15.4%) was not higher than that seen previously in patients ≥1 year of age;^20^ however, it should be noted that only a small number of patients were enrolled into this study and eligible for genotyping. One patient with influenza A/H1N1pdm09 infection was identified as having a PA/I38T substitution, which has previously been described,^26,27^ including in patients 1–<12 years of age.^20,28^ Omoto et al.^27^ previously reported that this substitution can confer reduced susceptibility to baloxavir; however, in the Phase III miniSTONE-2 study, clinical benefit was still observed in patients 1–<12 years old with substitutions at position I38 in PA (including I38T).^20,28^ In the current study, another patient was identified as having a novel PA/T20K substitution. The TTAS for the patients with PA/I38T and PA/T20K substitutions was approximately 3.0 days (72.2 hours) and 3.7 days (89.0 hours), respectively; the TTAS for the overall ITTi population (N = 15) was approximately 7 days (163.7 hours). Whilst the patient with a PA/I38T substitution experienced a viral rebound on Day 6, viral rebound was also recorded in 2 other patients without resistance. Therefore, in this small group of patients eligible for genotyping, resistance did not result in poorer clinical outcomes compared with the ITTi population.

### Limitations

The small patient population of this study (N = 48 for the safety population), designed primarily to evaluate PK and safety, may limit the reliability of the efficacy conclusions. Only a small number of patients had central laboratory-confirmed influenza, resulting in a small ITTi population of 15 patients. Additionally, only 1 enrolled patient was below the age of 4 weeks (Cohort III); therefore, safety conclusions for this subgroup could not be made. This study had no control arm; therefore, the results recorded were observational and qualitative in nature.

## CONCLUSIONS

Baloxavir was well tolerated among children <1 year old, with no new safety signals identified. Clinical, virological and safety outcomes were generally consistent with established profiles in adults, adolescents and children 1–<12 years of age. This study expands upon previous findings in older children and presents baloxavir as a potential new therapeutic option for children <1 year old. The single-dose oral regimen of baloxavir has the potential to enhance accessibility of antiviral treatment for this patient population.
